# Exploring the optimal regimen in advanced hepatocellular carcinoma: a protocol of individual patient data network meta-analysis of randomized controlled trials

**DOI:** 10.3389/fimmu.2026.1804661

**Published:** 2026-03-31

**Authors:** Jiefeng Luo, Zhengxiong Li, Jun Ge, Sizhen Cai, Xinyi Mo, Wensheng Liu, Chengxia Gui, Qiong Du

**Affiliations:** 1 Department of Pharmacy, Fudan University Shanghai Cancer Center, Shanghai, China; 2 Department of Oncology, Shanghai Medical College, Fudan University, Shanghai, China; 3 School of Medical Informatics and Engineering, Xuzhou Medical University, Xuzhou, China; 4 School of Chemistry, Sun Yat-sen University, Guangzhou, China; 5 School of Pharmacy, Guangzhou Medical University, Guangzhou, China; 6 Department of Internal Medicine, Sichuan Electric Power Hospital, Chengdu, China

**Keywords:** hepatocellular carcinoma, immunotherapy, individual patient data, network meta-analysis, tyrosine kinase inhibitors

## Abstract

**Background:**

Advanced hepatocellular carcinoma (HCC) poses a substantial global disease burden. Since the approval of sorafenib in 2007, an increasing number of treatment regimens have demonstrated encouraging survival benefits in first-line treatment of advanced HCC. However, this expansion of therapeutic options has also introduced complexity into clinical decision-making. This study aims to provide high-quality evidence to inform clinical practice by comparing all first-line treatment regimens for advanced HCC.

**Methods:**

We will systematically search PubMed, Embase (Ovid), and the Cochrane Library (Ovid) from January 1, 2007, to March 3, 2026. Supplementary searches will be performed in clinical trial platforms and conference abstracts. All randomized controlled trials (RCTs) and high-quality observational studies (for prior information only) comparing active first-line treatment regimen for advanced HCC will be included. Primary outcomes are overall survival, grade ≥3 serious adverse events, and the incremental safety-effectiveness ratio. Secondary outcomes include progression-free survival, objective response rate, the incidence of all adverse events and treatment discontinuation due to adverse events. The risk of bias of included RCTs will be assessed using the Risk of Bias 2.0 tool, and the certainty of evidence for each outcome will be evaluated with the Confidence in Network Meta-Analysis (CINeMA) application. Study selection, data extraction, and quality assessment will be performed independently by two reviewers, with any disagreements adjudicated by a third reviewer. Individual patient data will be reconstructed from published Kaplan-Meier curves, and treatment effects will be evaluated using restricted mean survival time. Subgroup analyses will be performed based on PD-L1 expression, etiology, Barcelona Clinic Liver Cancer stage, alpha-fetoprotein concentration, macrovascular invasion, and extrahepatic spread. Bayesian network meta-analysis will be performed using R with the gemtc package.

**Discussion:**

Our study is expected to inform clinical guidelines and support personalized therapeutic decisions for advanced HCC.

**Clinical trial registration:**

https://www.crd.york.ac.uk/PROSPERO/view/, identifier CRD420251126975.

## Introduction

Liver cancer represents a major global public health challenge. It is the sixth most commonly diagnosed cancer and the third leading cause of cancer-related death worldwide (1). The disease burden of liver cancer is substantial, with approximately 866,136 new cases and 758,725 deaths estimated globally in 2022 (2). In the United States (US), the annual medical cost for liver cancer is estimated to be as high as $454.9 million (3). This financial toxicity affecting a considerable proportion of patients, as 22.6% report an inability to afford their care (4). Hepatocellular carcinoma (HCC) is the dominant histologic subtype of primary liver cancer, accounting for more than 80% of all cases (5). The key risk factors of HCC including viral hepatitis (hepatitis B and C) and non-viral etiologies such as alcohol-associated liver disease and metabolic dysfunction-associated steatotic liver disease (1, 5).

Despite declining incidence and mortality rates in several traditionally high-burden regions, the trends in many European and North American countries have either plateaued at high levels or are rising (6). This shift indicates a transition of HCC from regions with low-to-middle sociodemographic indices to those with higher indices (1, 7). Data from the US shows that between 2000 and 2020, the incidence of HCC rose from 2.38 to 3.09 per 100,000 women and from 7.32 to 9.82 per 100,000 men (8). If the current trends persist, the annual number of new HCC cases is projected to reach 1.52 million by 2050, which would double the number recorded in 2022 (5).

For early-stage HCC, treatment options include radical therapies such as resection or transplantation for eligible patients, and locoregional control achievable through ablation or transarterial therapies like transarterial chemoembolization (5). Unfortunately, only 30–40% of HCC cases are detected at an early stage, with the majority presenting as advanced disease at initial diagnosis (5, 9). For these patients with advanced HCC, systemic therapy forms the cornerstone of management (1, 5). The goal of systemic therapy is to control disease progression, prolong overall survival (OS), and achieve partial or complete tumor remission in some patients (10).

The treatment paradigm for advanced HCC was fundamentally reshaped with the advent of small-molecule tyrosine kinase inhibitors (TKIs) (10). The landmark SHARP trial in 2007 established sorafenib as the first systemic therapy to demonstrate a significant OS benefit, becoming the standard of care for over a decade (11). This breakthrough was followed by lenvatinib, which proved non-inferior to sorafenib in the REFLECT trial (12). The subsequent approval of second-line treatments such as regorafenib (RESORCE trial) (13), cabozantinib (CELESTIAL trial) (14) further solidified the important role of TKIs in advanced HCC.

The remarkable success of immune checkpoint inhibitors (ICIs) in delivering substantial survival benefits across multiple cancer types (15, 16), they were initially considered a promising approach for advanced HCC. However, pivotal phase III trials of pembrolizumab and nivolumab demonstrated that ICI monotherapy failed to achieve a significant OS benefit over sorafenib (17, 18). This indicates that monotherapy immunotherapy is inadequate and there is an urgent need for more effective treatment strategies.

The treatment landscape for advanced HCC underwent a paradigm shift in 2020 with the epoch-making success of the IMbrave150 trial (19). This study demonstrates that atezolizumab plus bevacizumab has advantages over sorafenib in OS and progression-free survival (PFS), establishing atezolizumab plus bevacizumab as a new first-line standard. This strategy was rapidly reinforced by a series of subsequent positive randomized controlled trials (RCTs). These included sintilimab plus a bevacizumab biosimilar (ORIENT-32) (20), camrelizumab plus rivoceranib (CARES-310) (21), and anlotinib plus penpulimab (APOLLO) (22), all of which achieved significant improvements in OS and PFS. It is worth noting that the OS benefits of pembrolizumab plus lenvatinib (LEAP-002) (23) and atezolizumab plus cabozantinib (COSMIC-312) (24) did not meet expectations, which may remind us that not all ICI plus TKI are more effective than TKIs monotherapy.

Tremelimumab plus durvalumab demonstrated remarkable long-term survival, with a 31% OS rate at 48 months (25), while nivolumab plus ipilimumab achieved a median OS of 23.7 months (26). The long-term survival benefits and durable efficacy of dual ICI therapy make it a highly attractive treatment option. However, the increased risk of severe immune-mediated toxicity of dual ICI has caused great concern among clinical decision-makers.

## Rational

The therapeutic landscape for advanced HCC has evolved dramatically, transitioning from a scarcity of options to an abundance of first-line regimens. While this expansion represents significant progress, it simultaneously introduces considerable complexity into clinical decision-making. Determining the optimal treatment option that balances efficacy and safety remains controversial due to the lack of direct comparisons between different treatment regimens.

Network meta-analysis (NMA) serves as a valuable methodology for evaluating relative treatment effects when direct comparative evidence is lacking (27). Despite the publication of many NMAs in recent years, they remain constrained by several important methodological limitations (28–31). First, their reliance on the hazard ratio (HR) as the primary measure of survival benefit may be inadequate for capturing the unique efficacy profile of immunotherapies. The “delayed separation” or “long-term benefit” characteristic of survival curves with immunotherapies often leads to non-proportional hazards, violating a key assumption of the Cox model from which HRs are derived (32). Second, most existing NMAs report efficacy and safety outcomes respectively, without formally integrating both dimensions to evaluate the trade-offs that are fundamental to clinical practice.

Ciro Celsa and colleagues addressed the methodological limitations of HR by employing restricted mean survival time (RMST), a robust statistical approach that does not require meeting the proportional hazard assumption (33). Furthermore, they pioneered the use of the incremental safety-effectiveness ratio (ISER) to quantitatively balance survival gains against the risk of severe adverse events (SAE). However, the clinical landscape has since been reshaped by the recent publication of pivotal trials such as CheckMate 9DW (nivolumab plus ipilimumab) (26) and CARES-310 (camrelizumab plus rivoceranib) (21). The CheckMate 9DW study is particularly important because it was the first study to demonstrate superior efficacy compared to a control group primarily composed of lenvatinib (85%).

Therefore, an updated and methodologically rigorous NMA is urgently needed. This will provide crucial evidence for clinical decision-makers.

## Methods and analysis

Our study will be conducted in strict accordance with the recommendations of the Cochrane Handbook (34), and its reporting will adhere to the Preferred Reporting Items for Systematic Reviews and Meta-Analyses extension for Network Meta-Analyses (PRISMA-NMA) statement (35). The development of this protocol strictly follows the PRISMA for Protocols (PRISMA-P) statement (**Supplementary Materials Appendix I**) and has been prospectively registered on the international prospective register of systematic reviews (PROSPERO) with registration number CRD420251126975.

### Type of patients

Adult patients (age ≥18 years) with histologically confirmed advanced HCC will be included. Eligible patients must be deemed unsuitable for curative-intent surgical or locoregional therapies and must not have received any prior systemic anticancer treatment. No restrictions will be applied based on patient nationality, geographic region, sex, race, etiology, Eastern Cooperative Oncology Group (ECOG) performance status, alpha-fetoprotein (AFP) concentration, presence of macrovascular invasion (MVI), or Barcelona Clinic Liver Cancer (BCLC) stage.

### Type of studies

All available Phase II and Phase III RCTs will be included, with no restrictions on publication language. We will translate non-English documents into English. If multiple publications are published for the same RCT, select the publication that provides the most comprehensive data or the longest follow-up period. In addition, to provide prior information for the Bayesian network meta-analysis of RCTs, we also included high-quality observational studies.

### Types of interventions

Eligible RCTs must include at least two of the following treatment regimens: TKI monotherapy, ICI monotherapy, ICI plus TKI, dual ICI, dual ICI plus TKI, placebo, standard of care, or any other available active therapy.

### Types of outcome measures

#### Primary outcomes

The primary outcomes of this study are OS, the incidence of grade ≥3 SAE, and the ISER. ISER has demonstrated in previous studies that it can combine efficacy and safety into a single net clinical benefit measure (33). It is calculated by dividing the absolute difference in SAE rates between two treatment regimens by their absolute difference in OS or PFS, measured in months. The results represent the percentage increase in the number of patients experiencing SAEs for each additional month of survival. To assess the robustness of this ISER, a two-way sensitivity analysis will be conducted by varying both the numerator (difference in SAE rates) and the denominator (gain in life-months) within their respective confidence intervals (CIs). Interpreting the ISER requires defining a “willingness-to-risk” threshold, like the “willingness-to-pay” threshold in cost-effectiveness analyses. This threshold establishes the acceptable level of additional risk for a given unit of survival benefit. Unfortunately, unlike willingness-to-pay threshold are commonly used, no such consensus exists for SAE risk tolerance. Therefore, we will present ISER results across a range of plausible willingness-to-risk thresholds and show how the preferred treatment changes accordingly. In clinical practice, decision-makers can engage patients in a trade-off discussion by asking, “How much risk of a SAE would you be willing to accept for an additional month of progression-free survival?” Based on the patient’s stated acceptable risk level, and referring to the corresponding preferred treatment probabilities across our pre-specified range of thresholds, the most suitable regimen for that individual patient can be identified.

#### Secondary outcomes

Secondary outcomes will include PFS, the objective response rate (ORR), the incidence of all AEs and the treatment discontinuation rate due to AEs.

### Information source and search strategy

We will conduct systematic search through PubMed, Embase (Ovid), and the Cochrane Library (Ovid). The search will cover the period from January 1, 2007, to March 3, 2026, to encompass all systemic therapy for HCC beginning with the approval of sorafenib. To ensure comprehensiveness and minimize publication bias, we will also search the following clinical trial platforms: ClinicalTrials.gov, the Chinese Clinical Trial Registry, and the WHO International Clinical Trials Registry Platform. Additionally, to identify unpublished but potentially significant data, conference abstracts from the European Society for Medical Oncology (ESMO), the American Society of Clinical Oncology (ASCO), and the Chinese Society of Clinical Oncology (CSCO) will be reviewed. The search strategy will combine Medical Subject Headings (MeSH) with free text terms. Key search terms will include: “liver cancer”, “hepatocellular carcinoma”, “tyrosine kinase inhibitor”, “vascular endothelial growth factor inhibitor”, and “immune checkpoint inhibitor”. **Table 1** details our search strategy on PubMed. The search strategies for Embase (OVID) and Cochrane Library (Ovid) will be presented in the **Supplementary Materials Appendix II–III**.

**Table 1:** Search strategy on PubMed.

Search strategy
((“Carcinoma, Hepatocellular”[Mesh] OR “Liver Neoplasms”[Mesh] OR hepatocellular carcinoma[tiab] OR HCC[tiab] OR liver cancer[tiab] OR liver neoplasm*[tiab]) AND (“Antineoplastic Combined Chemotherapy Protocols”[Mesh] OR “Immune Checkpoint Inhibitors”[Mesh] OR “Angiogenesis Inhibitors”[Mesh] OR “Protein Kinase Inhibitors”[Mesh] OR atezolizumab[tiab] OR bevacizumab[tiab] OR sintilimab[tiab] OR camrelizumab[tiab] OR rivoceranib[tiab] OR apatinib[tiab] OR durvalumab[tiab] OR tremelimumab[tiab] OR nivolumab[tiab] OR ipilimumab[tiab] OR pembrolizumab[tiab] OR lenvatinib[tiab] OR sorafenib[tiab] OR regorafenib[tiab] OR cabozantinib[tiab] OR “TKIs”[tiab] OR “tyrosine kinase inhibitor*”[tiab])

### Study selection

All publications will be imported into EndNote X9 (Clarivate Analytics, Philadelphia, PA, USA) for duplicate removal. Subsequently, two independent reviewers (Jiefeng Luo and Zhengxiong Li) will perform the study selection in two phases. First, they will screen the titles and abstracts of all publications, excluding those that are obviously irrelevant. Second, they will independently assess the full texts of the remaining publications to determine final eligibility for inclusion. Any disagreements between the reviewers will be resolved through discussion, and if a consensus cannot be reached, a third reviewer (Qiong Du) will make the final adjudication. The entire study selection process will be presented in a PRISMA flow diagram.

### Data collection

A standardized data extraction form will be developed in advance. To ensure consistency, a pilot extraction involving approximately 10% of the included RCTs will be independently performed by two reviewers (Sizhen Cai and Xinyi Mo), with the results reviewed by a third reviewer (Jun Ge) to calibrate the process. The form will capture comprehensive data including basic study information (title, authors, publication year, journal), methodological information (country/region, number of centers, randomization, blinding, allocation concealment, study dates, eligibility criteria, sample size), patient information (age, race, sex, etiology, ECOG performance status, Child–Pugh score, BCLC stage, MVI, AFP concentration, extrahepatic spread), intervention information and outcomes. The above extraction process will be completed independently by two reviewers (Sizhen Cai and Xinyi Mo) (36, 37). If there are any disputes that cannot reach a consensus, a third reviewer (Jun Ge) will make the decision. For outcomes, OS is defined as the time from randomization to death from any cause. PFS is defined as the time from randomization to the first documented disease progression, as assessed by blinded independent central review according to RECIST version 1.1, or death from any cause, whichever occurs first. The ORR is defined as the proportion of patients achieving a complete or partial response per RECIST 1.1. If any data is missing, we will contact the corresponding author via email.

### Risk of bias and evidence certainty assessment

In accordance with the Cochrane Handbook for Systematic Reviews of Interventions, the risk of bias for each included RCT will be independently assessed by two reviewers (Wensheng Liu and Chengxia Gui) using the revised Cochrane Risk of Bias tool for randomized trials (RoB 2). Any disagreements in assessment that cannot be resolved through discussion will be adjudicated by a third reviewer (Jiefeng Luo). The RoB 2 tool categorizes each RCT as having a “low,” “some concerns” or “high” risk of bias based on the bias assessment results across five domains (38, 39). Inter-rater agreement between the two reviewers will be quantified using Cohen’s kappa coefficient. If the kappa value < 0.8, the two reviewers will discuss identifying the main sources of disagreement, and a third reviewer will adjudicate if necessary.

The certainty of evidence for each outcome will be independently assessed by two reviewers (Wensheng Liu and Chengxia Gui) using the Confidence in Network Meta-Analysis (CINeMA) application, which is based on the Grading of Recommendations Assessment, Development and Evaluation (GRADE) framework (40). Any disagreements in judgment that cannot be resolved through discussion will be adjudicated by a third reviewer (Jiefeng Luo). The CINeMA application evaluates the certainty of evidence across six key domains: within-study bias, reporting bias, indirectness, imprecision, heterogeneity, and incoherence.

### Individual patient survival data extraction

To derive time-to-event data, we will digitally extract coordinate points from the published Kaplan-Meier (KM) curves for OS and PFS in all included RCTs using the automated extraction software SurvdigitizeR (https://pechlilab.shinyapps.io/SurvdigitizeR/) (41). The established algorithm by Guyot et al. will then be applied to convert these data points into reconstructed pseudo-individual patient data, from which both a KM curve and a corresponding risk table (containing survival time, number of patients at risk, and number of events) will be generated for each study (42–44). To validate the accuracy of this process, we will compare the reconstructed KM curves against the original curves and assess the concordance between the reported and reconstructed median OS and PFS. The validation criteria include: (1) consistency of the number of patients at risk at time zero (randomization) with the published data; and (2) an absolute difference in median OS/PFS between the reconstructed and published data of no more than 0.2 months. If any of these criteria are not met, we will repeat the extraction and reconstruction steps until the validation standards are satisfied. Furthermore, to assess the robustness of the reconstruction process, we will perform a sensitivity analysis using an alternative software for graphical data extraction(WebPlotDigitizer) to manually digitize the same KM curves and reconstruct the individual patient data.

### Data synthesis

To avoid potential non-proportional hazards in the included RCTs, we will use the RMST as the primary statistical measure of treatment effect. RMST represents the average survival time within a specified, clinically relevant time window and is calculated as the area under the KM survival curve up to a pre-defined timepoint. For PFS, a 12-month timeframe will be used; for OS, 24-month and 36-month timeframes will be used (45). Continuous outcomes will be summarized using mean difference (MD) and its 95% CI, and dichotomous outcomes will be summarized using OR and its 95% CI. The surface under the cumulative ranking curve (SUCRA) will be used to rank all treatments regimens for each outcome (27).

The NMA will be performed within a Bayesian framework using Markov chain Monte Carlo (MCMC) methods. Specifically, analyses will be conducted in R 4.5.1 using the gemtc package (46). We will run 6 Markov chains with an initial adaptation phase of 50,000 iterations, followed by a simulation phase of 200,000 iterations, applying a thinning factor of 10. The choice between fixed effects model and random effects model will be guided by the deviance information criterion (DIC). A random effects model will be selected if the difference in DIC is less than 3; otherwise, the model with the lower DIC will be chosen (27, 47).

Global heterogeneity will be assessed using the χ² test and quantified by the *I²* statistic. *I²*≤25% will be considered low heterogeneity, 25%<*I²*≤75% will be considered moderate heterogeneity, and 75%<*I²* will be considered high heterogeneity (48). We plan to use the posterior distribution of the heterogeneity variance τ^2^ to quantify its uncertainty and present prediction intervals to illustrate the impact of heterogeneity on the effect estimates. Potential covariates that may affect transitivity will be presented in tabular form. In addition, we plan to conduct network meta-regression to explore the relationship between these covariates and effect sizes. Consistency between direct and indirect evidence within closed loops of the network will be evaluated using the node-splitting approach. The network diagram of included RCTs will be visualized using the CINeMA application, while the gemtc package will be utilized to create league tables and forest plots. Publication bias will be assessed by funnel plots, Begg’s test and Egger’s regression (48).

To address the potential sparsity of data for some interventions and to ensure the stability of network estimates, we will incorporate external evidence from high-quality observational studies (49). In detail, we will include large observational studies (sample size >200 patients) that evaluate the first-line regimens of interest. We will perform a separate meta-analysis of the observational studies to obtain a pooled effect estimate μ_obs_ and its variance σ^2^_obs_​. This pooled result will be used to construct an informative prior distribution for the corresponding treatment effect parameter in the main Bayesian network meta-analysis of RCTs. The prior will be specified as dk∼N(μ_obs_, σ^2^_obs_×*w*), where *w* is a downweighting factor greater than 1. To account for the inherent biases and higher uncertainty associated with observational data, we will downweighting their influence by inflating the prior variance and will conduct a series of sensitivity analyses using different downweighting factors. This approach allows the observational data to inform the model for sparse interventions while ensuring that the primary conclusions remain anchored in the higher-quality RCT evidence.

For interventions with sparse RCT evidence, informative priors will be constructed from meta-analyses of observational studies, specified as dk∼N(μ_obs_, σ^2^_obs_×*w*), where w is a downweighting factor greater than 1. For all other interventions, the default vague prior in gemtc will be used: *d*∼*N*(0,re.prior.sd^2^).

For the between-study heterogeneity parameter (τ), we will employ empirically-derived informative priors based on the predictive distributions from Turner et al., which were developed from 14,886 Cochrane meta-analyses. Priors are selected according to outcome type: (1) OS (all-cause mortality): log normal prior: τ∼logN(−4.06,1.452), (2) PFS and ORR (semi objective outcomes): log normal prior: τ∼logN(−3.02,1.852), (3) AEs and SAE(subjective outcomes): log normal prior: τ∼logN(−1.87,1.522) (50, 51).

### Subgroup analysis

The purpose of the subgroup analyses is to stratify patients and inform personalized treatment strategies by identifying optimal treatment regimens within specific subgroups. The predefined subgroups include PD-L1 expression (combined positive score [CPS] ≥1; CPS <1), etiology (hepatitis B, hepatitis C, non-viral), BCLC stage (BCLC B, BCLC C), AFP concentration (<400 μg/L, ≥400 μg/L), MVI (yes, no), and presence of extrahepatic spread (yes, no). It is worth noting that all of our subgroup analyses were hypothesis-driven.

For RCTs that provide KM curves for specific subgroups, we will reconstruct pseudo-individual patient data from these curves to conduct an individual patient data-based NMA. For RCTs where subgroup KM curves are unavailable but a subgroup HR with its CI is reported, we will perform an aggregate data NMA.

To maximize data utilization and address missing subgroup KM curves, we will implement the KMSubtraction method proposed by Zhao et al. (52). This method mathematically derives the KM curve for an unreported subgroup by subtracting the KM curve of its complementary reported subgroup from the overall trial KM curve, thereby reconstructing otherwise missing subgroup time-to-event data.

### Patient and public involvement

There was no patient or public involvement in this work.

## Discussion

While the post-atezolizumab plus bevacizumab era has witnessed an increasing number of ICI-based combinations demonstrating benefit in first-line advanced HCC, the ensuing debate over the optimal regimen has intensified. The 2025 National Comprehensive Cancer Network (NCCN) guidelines include nivolumab plus ipilimumab as a first-line advanced HCC option but assigns it a Category 2A recommendation which is a lower strength of recommendation compared to the Category 1A recommendations for atezolizumab plus bevacizumab and durvalumab plus trimemumab (53). Similarly, the 2025 European Society for Medical Oncology (ESMO) guidelines list camrelizumab plus rivoceranib and nivolumab plus ipilimumab as first-line advanced HCC therapies(I, B) but with a lower evidence grade than the recommendations for atezolizumab plus bevacizumab and durvalumab plus tremelimumab (I, A) (54). This difference may reflect a lack of long-term real-world safety and efficacy data for newly approved regimens, as well as a key evidence gap in comparisons with established standard treatments. Therefore, our study aims to provide timely and crucial evidence to inform this complex clinical decision-making landscape.

Our study has several key strengths. First, it incorporates the latest pivotal trials that may reshape the current treatment paradigm. Second, by reconstructing individual patient data and employing the RMST as our effect measure, we can more accurately capture the long-term survival benefits of immunotherapies without considering the proportional hazards assumption. Third, the integration of efficacy and safety through the ISER offers clinical decision-makers an intuitive metric for evaluating therapeutic trade-offs. Fourth, we will apply the KMSubtraction method to reconstruct unreported subgroup KM curves which allows for a more granular analysis than previous NMAs. Fifth, the inclusion of Phase II RCTs alongside Phase III RCTs enriches our assessment of the ORR, which may offer valuable insights for future clinical development. Sixth, incorporating observational data as informative priors for treatment effects and empirically derived distributions for heterogeneity enhances the robustness of network estimates by stabilizing sparse comparisons and improving between-study variance estimation.

We also acknowledge the limitations of our study. First, for interventions reporting only ORR without survival data, we will be unable to conduct a unified benefit-risk assessment of safety and efficacy. Second, our individual patient data reconstruction from published KM curves does not grant access to individual patient baseline characteristics, precluding an analysis of treatment-effect interactions with key covariates. Third, potential heterogeneity may be introduced by temporal trends in the efficacy of the same treatment regimen over different trial periods (5). Sorafenib reported a median OS increase from 10.7 months in the SHARP trial (11) to 13–15 months in recent RCTs (20, 21, 25). A similar trend was observed with lenvatinib, with its median OS extending from 13.6 months in the REFLECT trial (12) to 19.8 months in the CheckMate 9DW trial (26). This gradual improvement in survival benefits is unlikely to be attributable to the drugs, but a more plausible explanation is a shift in patient management practices, including earlier initiation of systemic therapy and more refined supportive care (5). Fourth, the ISER lacks a universally accepted willingness-to-risk threshold. We will present the probability that each regimen is preferred across a range of plausible risk levels to inform individualized decision-making.
