# Supplementary material for: Exploring the optimal regimen in advanced hepatocellular carcinoma: a protocol of individual patient data network meta-analysis of randomized controlled trials
Source: Front Immunol. 2026 Mar 31;17:1804661. doi: 10.3389/fimmu.2026.1804661 (PMC13076546; doi:10.3389/fimmu.2026.1804661)
Supplement: Supplementary file 1 [file Table1.docx]

**Supplementary Materials**

**Appendix I**

**PRISMA-P (Preferred Reporting Items for Systematic review and Meta-Analysis Protocols) 2015 checklist: recommended items to address in a systematic review protocol***

| Section and topic | Item | Checklist item | Report |
| --- | --- | --- | --- |
| ADMINISTRATIVE INFORMATION | | |  |
| Title: |  |  |  |
| Identification | 1a | Identify the report as a protocol of a systematic review | Yes |
| Update | 1b | If the protocol is for an update of a previous systematic review, identify as such | NA |
| Registration | 2 | If registered, provide the name of the registry (such as PROSPERO) and registration number | Yes |
| Authors: |  |  |  |
| Contact | 3a | Provide name, institutional affiliation, e-mail address of all protocol authors; provide physical mailing address of corresponding author | Yes |
| Contributions | 3b | Describe contributions of protocol authors and identify the guarantor of the review | Yes |
| Amendments | 4 | If the protocol represents an amendment of a previously completed or published protocol, identify as such and list changes; otherwise, state plan for documenting important protocol amendments | NA |
| Support: |  |  |  |
| Sources | 5a | Indicate sources of financial or other support for the review | Yes |
| Sponsor | 5b | Provide name for the review funder and/or sponsor | NA |
| Role of sponsor or funder | 5c | Describe roles of funder(s), sponsor(s), and/or institution(s), if any, in developing the protocol | NA |
| INTRODUCTION | | |  |
| Rationale | 6 | Describe the rationale for the review in the context of what is already known | Yes |
| Objectives | 7 | Provide an explicit statement of the question(s) the review will address with reference to participants, interventions, comparators, and outcomes (PICO) | Yes |
| METHODS | | |  |
| Eligibility criteria | 8 | Specify the study characteristics (such as PICO, study design, setting, time frame) and report characteristics (such as years considered, language, publication status) to be used as criteria for eligibility for the review | Yes |
| Information sources | 9 | Describe all intended information sources (such as electronic databases, contact with study authors, trial registers or other grey literature sources) with planned dates of coverage | Yes |
| Search strategy | 10 | Present draft of search strategy to be used for at least one electronic database, including planned limits, such that it could be repeated | Yes |
| Study records: |  |  |  |
| Data management | 11a | Describe the mechanism(s) that will be used to manage records and data throughout the review | Yes |
| Selection process | 11b | State the process that will be used for selecting studies (such as two independent reviewers) through each phase of the review (that is, screening, eligibility and inclusion in meta-analysis) | Yes |
| Data collection process | 11c | Describe planned method of extracting data from reports (such as piloting forms, done independently, in duplicate), any processes for obtaining and confirming data from investigators | Yes |
| Data items | 12 | List and define all variables for which data will be sought (such as PICO items, funding sources), any pre-planned data assumptions and simplifications | Yes |
| Outcomes and prioritization | 13 | List and define all outcomes for which data will be sought, including prioritization of main and additional outcomes, with rationale | Yes |
| Risk of bias in individual studies | 14 | Describe anticipated methods for assessing risk of bias of individual studies, including whether this will be done at the outcome or study level, or both; state how this information will be used in data synthesis | Yes |
| Data synthesis | 15a | Describe criteria under which study data will be quantitatively synthesised | Yes |
|  | 15b | If data are appropriate for quantitative synthesis, describe planned summary measures, methods of handling data and methods of combining data from studies, including any planned exploration of consistency (such as I^2^, Kendall’s τ) | Yes |
|  | 15c | Describe any proposed additional analyses (such as sensitivity or subgroup analyses, meta-regression) | Yes |
|  | 15d | If quantitative synthesis is not appropriate, describe the type of summary planned | NA |
| Meta-bias(es) | 16 | Specify any planned assessment of meta-bias(es) (such as publication bias across studies, selective reporting within studies) | Yes |
| Confidence in cumulative evidence | 17 | Describe how the strength of the body of evidence will be assessed (such as GRADE) | Yes |

*** It is strongly recommended that this checklist be read in conjunction with the PRISMA-P Explanation and Elaboration (cite when available) for important clarification on the items. Amendments to a review protocol should be tracked and dated. The copyright for PRISMA-P (including checklist) is held by the PRISMA-P Group and is distributed under a Creative Commons Attribution Licence 4.0.**

*From: Shamseer L, Moher D, Clarke M, Ghersi D, Liberati A, Petticrew M, Shekelle P, Stewart L, PRISMA-P Group. Preferred reporting items for systematic review and meta-analysis protocols (PRISMA-P) 2015: elaboration and explanation. BMJ. 2015 Jan 2;349(jan02 1):g7647.*

**Appendix II Search strategy on Embase (OVID)**

1 exp liver cell carcinoma/dt [Drug Therapy]

2 exp liver cell carcinoma/

3 liver tumor/

4 exp liver tumor/dt [Drug Therapy]

5 exp liver tumor/

6 "metastatic liver cancer".mp.

7 "advanced hepatocellular carcinoma".mp.

8 "advanced liver cancer".mp.

9 "metastatic hepatocellular carcinoma".mp.

10 ("metastatic HCC" or "advanced HCC").mp. [mp=title, abstract, heading word, drug trade name, original title, device manufacturer, drug manufacturer, device trade name, keyword, floating subheading word, candidate term word]

11 1 or 2 or 3 or 4 or 5 or 6 or 7 or 8 or 9 or 10

12 exp antineoplastic agent/

13 exp protein kinase inhibitor/

14 exp immunotherapy/

15 exp placebo/

16 exp drug therapy/

17 (antineoplastic* or "kinase inhibitor" or TKI or placebo*).mp. [mp=title, abstract, heading word, drug trade name, original title, device manufacturer, drug

manufacturer, device trade name, keyword, floating subheading word, candidate term word]

18 12 or 13 or 14 or 15 or 16 or 17

19 advanced.ti. or advanced.ab. or unresectable.ti. or unresectable.ab. or metastas*.ti. or metastas*.ab.

20 (first-line or second-line).mp. [mp=title, abstract, heading word, drug trade name, original title, device manufacturer, drug manufacturer, device trade name, keyword, floating subheading word, candidate term word]

21 exp survival/ or exp progression free survival/ or exp survival analysis/ or exp survival rate/

22 exp mortality/

23 exp treatment outcome/

24 21 or 22 or 23

25 11 and 19

26 18 and 25

27 24 and 26

28 20 and 27

**Appendix III Search strategy on Cochrane Library (Ovid)**

1 hepatocellular carcinoma.mp. [mp=title, short title, abstract, full text, keywords, caption text]

2 (unresectable or metastas* or metastat* or advanced).mp. [mp=title, short title, abstract, full text, keywords, caption text]

3 1 and 2

4 (antineoplastic* or PKI or protein kinase inhibitors or placebo).mp. [mp=title, short title, abstract, full text, keywords, caption text]

5 (first-line or second-line or "phase 3" or "phase III").mp. [mp=title, short title, abstract, full text, keywords, caption text]

6 3 and 4

7 5 and 6
